# Supplementary material for: Age-dependent appearance of SARS-CoV-2 entry sites in mouse chemosensory systems reflects COVID-19 anosmia-ageusia symptoms
Source: Commun Biol. 2021 Jul 15;4:880. doi: 10.1038/s42003-021-02410-9 (PMC8282876; doi:10.1038/s42003-021-02410-9)
Supplement: Supplementary file 2 — SUPPLEMENTAL INFORMATION [file 42003_2021_2410_MOESM2_ESM.pdf]

Supplementary Information

Age-dependent appearance of SARS-CoV-2 entry sites in mouse chemosensory systems reflects COVID-19 anosmia-ageusia symptoms.

Julien Brechbühl, Ana Catarina Lopes, Dean Wood, Sofiane Bouteiller, Aurélie de Vallière, Chantal Verdumo and Marie-Christine Broillet.

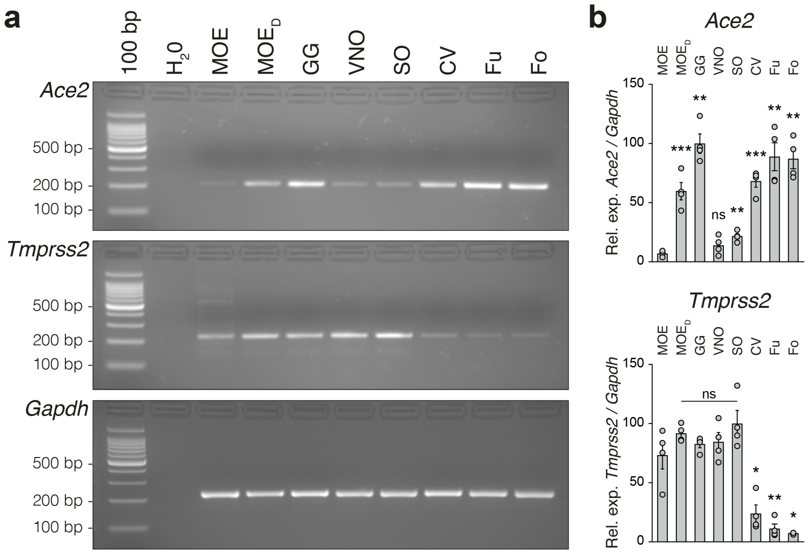

Supplementary Fig. 1 Semi-quantitative RT-PCR analysis of *Ace2* and *Tmprss2* in the mouse chemosensory systems. a Uncropped, unedited and unprocessed Figure 1b. **b** Gene expression profiles of the SARS-CoV-2 entry sites: *Ace2* and *Tmprss2* in the different mouse chemosensory systems performed by RT-PCR is here semi-quantified. Samples for gene expression profiles are obtained from 5-10 heterozygous OMP-GFP mice of 4 to 11 months old. Data are expressed relatively to the MOE and represented as mean ± SEM with aligned dot plots for $n = 4$ individual sample values. For comparisons between conditions, two-tailed Student's *t*-tests or Mann–Whitney tests are used, *p < 0.05, **p < 0.01, ***p < 0.001, ns for non-significant. Ladder of 100 base pairs (bp, (**a**)).

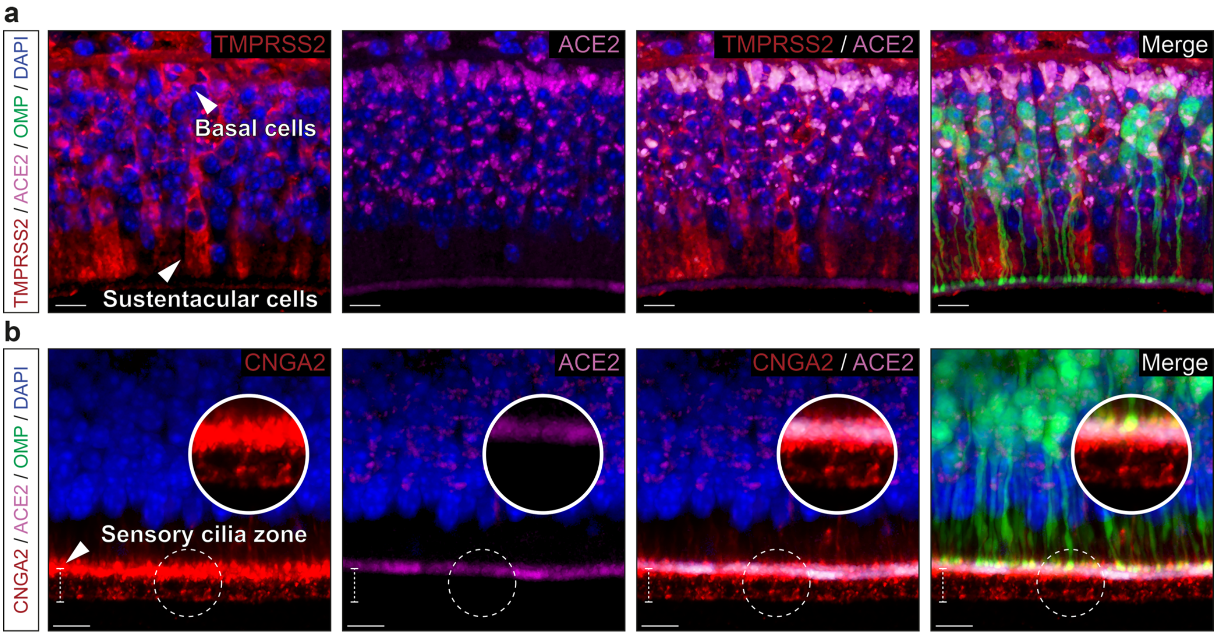

Supplementary Fig. 2 Detailed of the TMPRSS2 and ACE2 expression in the MOE$_{D}$. a TMPRSS2 (in red) is irregularly localized in different regions of the neuroepithelium. In addition to OMP- basal cell expression, sporadic cells express the TMPRSS2 protein. Here, TMPRSS2+ sustentacular cells co-stained with ACE2 (in pink) are shown. **b** Apical expression of ACE2 is found below the CNGA2+ sensory cilia zone (in red; highlighted by a dashed white line). Colocalization between red and pink signals is highlighted in light grey (**a**, **b**). Nuclei are counterstained with Dapi (DAPI, in blue). Representative protein expression profile obtained from heterozygous OMP-GFP mice of 7 (**a**) and 13 (**b**) months old. Scale bars are 10 μm.

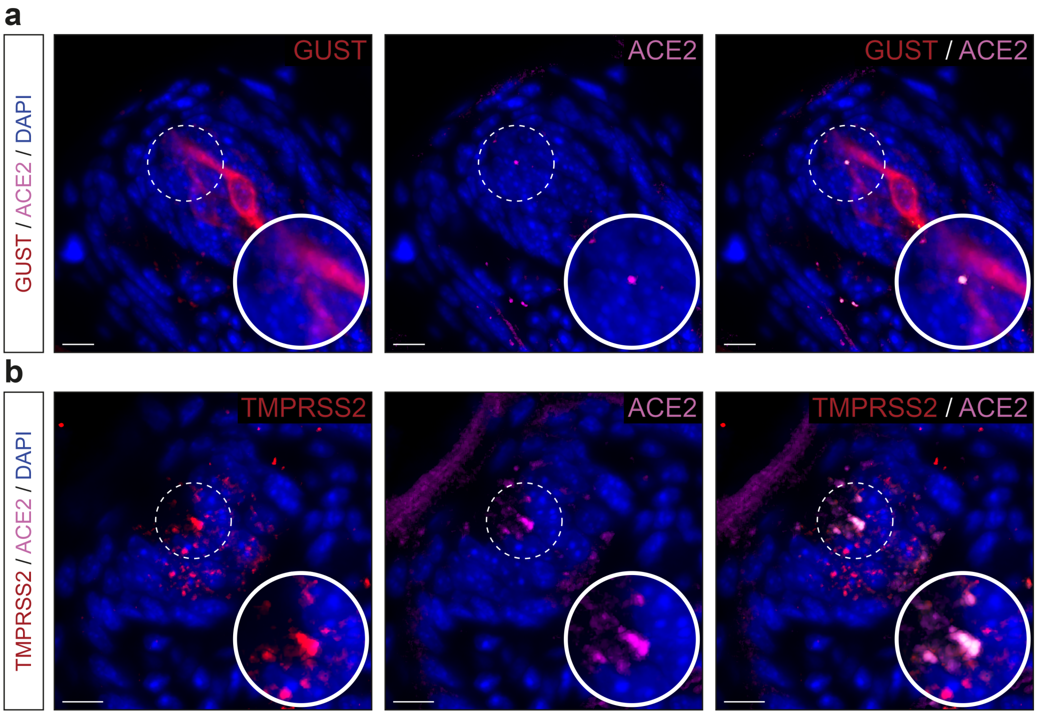

Supplementary Fig. 3 Expression profile of the ACE2 and TMPRSS2 proteins in taste fungiform papillae. Immunohistochemical investigations on the taste buds for SARS-CoV-2 entry sites in fungiform papillae. **a** The gustducin marker protein (GUST; in red) allows the localization of the gustatory sensory cells. ACE2 (in pink) expression in the microvilli of Gust+ sensory cells; a zoom in view of the microvilli of a taste pore is shown. **b** Co-expression profile of TMPRSS2 (in red) and ACE2 in the taste pores (highlighted in a zoom in view). Colocalization between red and pink signals is highlighted in light grey (**a**, **b**). Nuclei are counterstained with Dapi (DAPI, in blue). Representative protein expression profiles were obtained from heterozygous OMP-GFP mice of 5 (**a**) and 13 (**b**) months old. Scale bars are 10 μm (**a**, **b**).

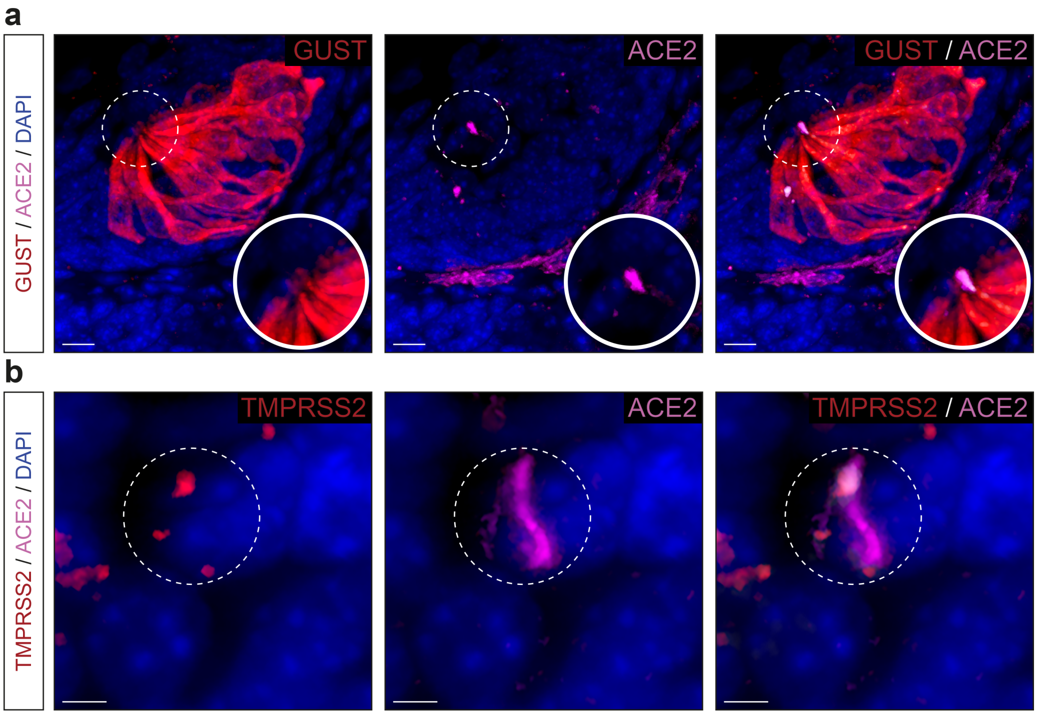

Supplementary Fig. 4 Expression profile of the ACE2 and TMPRSS2 proteins in taste foliate papillae. Immunohistochemical investigations on the taste buds for SARS-CoV-2 entry sites in foliate papillae. **a** The gustducin marker protein (GUST; in red) allows the localization of the gustatory sensory cells. ACE2 (in pink) expression in microvilli of Gust+ sensory cells; a zoom in view of the microvilli of a taste pore is shown. **b** Co-expression profile of TMPRSS2 (in red) and ACE2 in a taste pore is highlighted here. Colocalization between red and pink signals is highlighted in light grey (**a**, **b**). Nuclei are counterstained with Dapi (DAPI, in blue). Representative protein expression profiles were obtained from heterozygous OMP-GFP mice of 5 months old (**a**, **b**). Scale bars are 10 μm (**a**) and 3 μm (**b**).

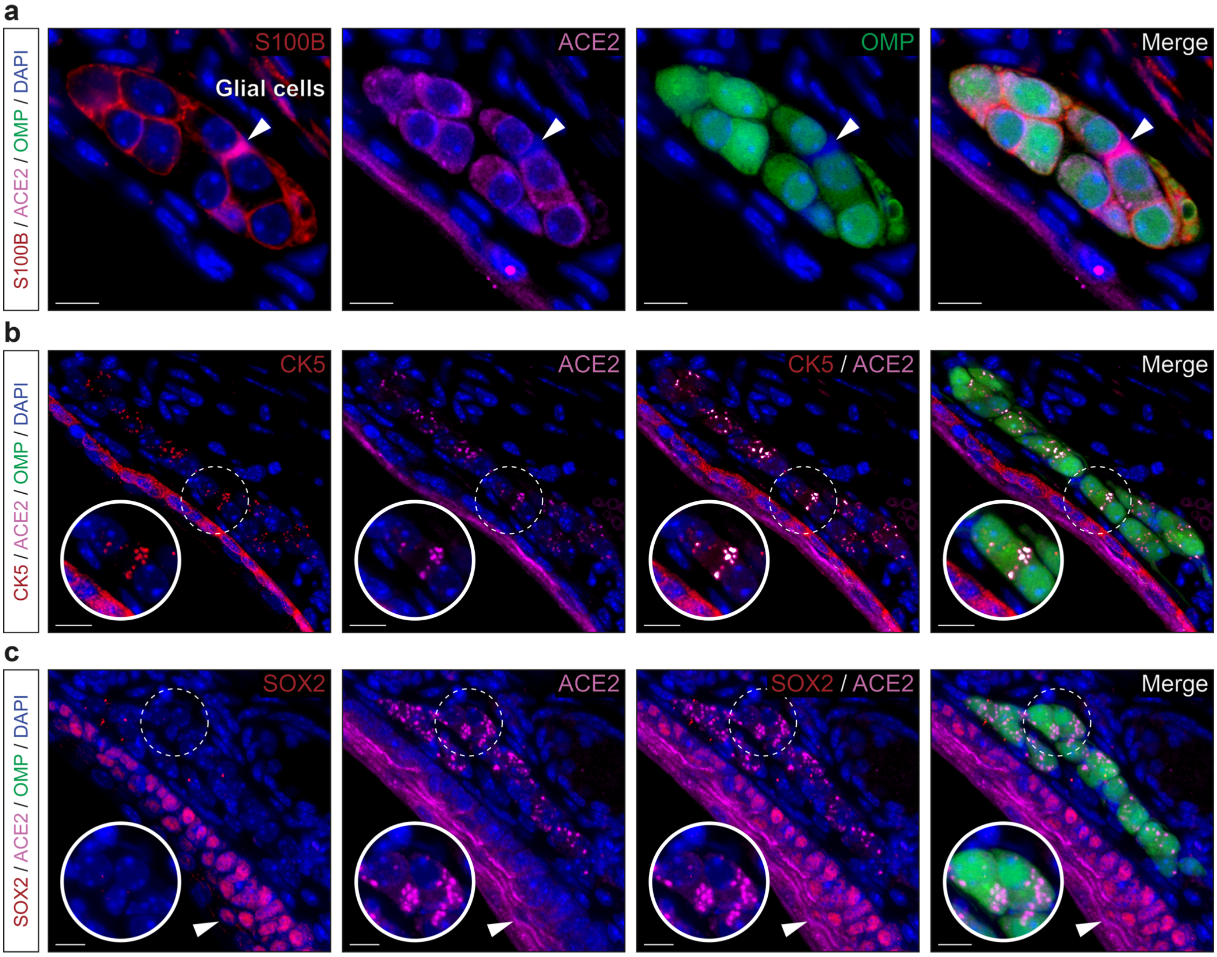

Supplementary Fig. 5 ACE2 is expressed in GG neurons and not in supporting glial cells. a ACE2 (in pink) is expressed in OMP+ GG neurons (in green) and absent in their surrounding S100B+ glial cells (in red). White arrow head highlights a precise wrapping glial cell where a clear absence of ACE2 staining is observed. **b** Co-expression of ACE2 with CK5+ punctiform staining in GG neurons (CK5, in red). Absence of co-expression in the apical keratinocyte cell layer (zoom in view, white dashed circle). **c** Co-expression of ACE2 and SOX2 (SOX2, in red) in apical keratinocyte cells (white arrowhead). Absence of SOX2 expression in OMP+ GG neurons (zoom in view, white dashed circle). Colocalization between red and pink signals is highlighted in light grey (**a**-**c**). Nuclei are counterstained with Dapi (DAPI, in blue). Representative protein expression profile obtained from heterozygous OMP-GFP mice of 13 (**a**), 6 (**b**) and 7 months old (**c**). Scale bars are 10 μm (**a**) and 15 μm (**b**, **c**).

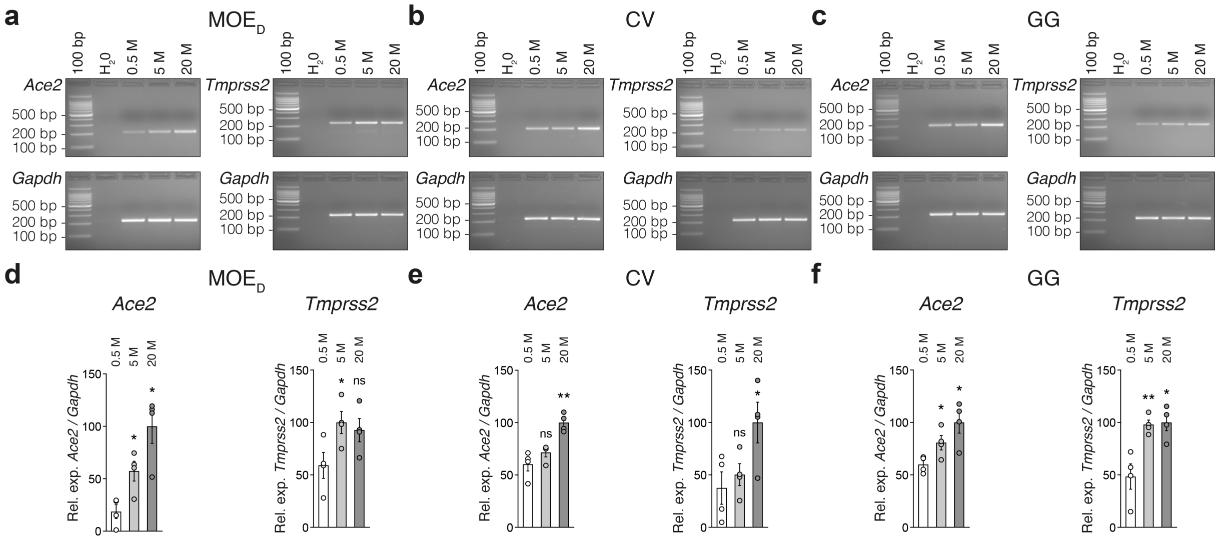

Supplementary Fig. 6 Semi-quantitative RT-PCR analysis of age-dependent expression of *Ace2* and *Tmprss2* in the MOE$_D$, CV and GG. a-c Uncropped, unedited and unprocessed Figure 8a-f. **d-f** RT-PCR semi-quantification of *Ace2* and *Tmprss2* in the MOE$_D$ (**d**), CV (**e**) and GG (**f**) at the indicated age in months (M). Samples for gene expression profiles are obtained from 5-6 heterozygous OMP-GFP mice. Data are expressed relatively to the 0.5 M and represented as mean ± SEM with aligned dot plots for n = 4 individual sample values. For comparisons between conditions, two-tailed Student's t-tests or Mann–Whitney tests are used, *p < 0.05, **p < 0.01, ns for non-significant. Ladder of 100 base pairs (bp, (**a**-**c**)).

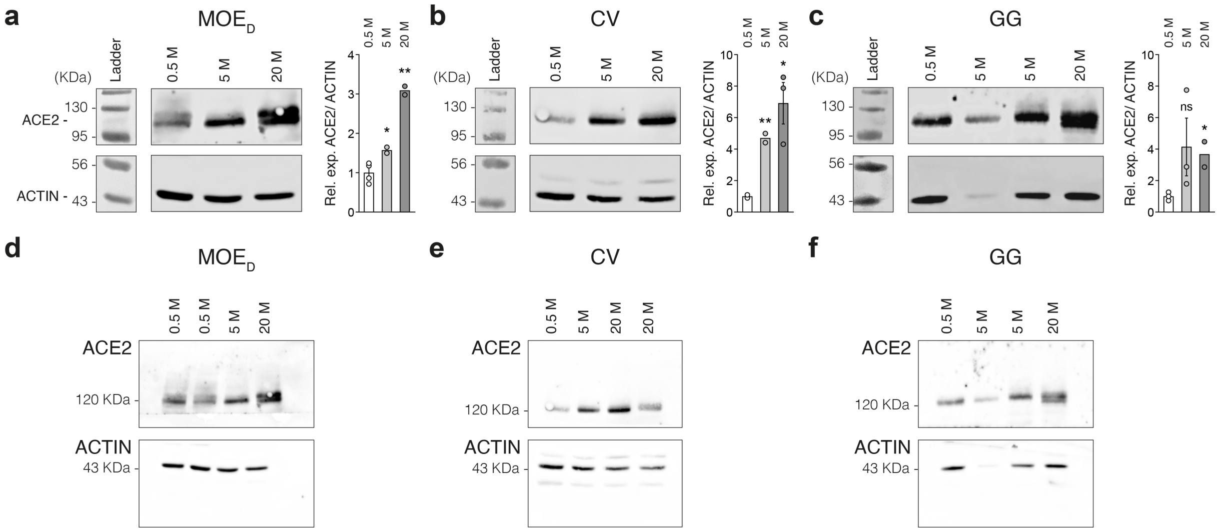

Supplementary Fig. 7 Western-blot analysis of the age-dependent expression of ACE2 in the MOE$_D$, CV and GG. a-c Western-blot analysis (left part of panels) and corresponding semi-quantifications (right part of panels) of ACE2 expression in the MOE$_D$ (**a**), CV (**b**) and GG (**c**) at the indicated age in months (M). **d-f** Uncropped, unedited and unprocessed Supplementary Figure 7a-c. Loading blots are separated for ACE2 and ACTIN antibody chemoluminescent detection. ACTIN is used as a house-keeping control. Samples for protein expression profiles are obtained from 2-6 heterozygous OMP-GFP mice. Data are expressed relatively to the 0.5 M and represented as mean ± SEM with aligned dot plots for *n* = 2-4 individual sample values. For comparisons between conditions, two-tailed Student's *t*-tests or Mann–Whitney tests are used, *p < 0.05, **p < 0.01, ns for non-significant. Protein Ladder (KDa, (**a**-**c**)).
