# Supplementary material for: Age-dependent appearance of SARS-CoV-2 entry sites in mouse chemosensory systems reflects COVID-19 anosmia-ageusia symptoms
Source: Commun Biol. 2021 Jul 15;4:880. doi: 10.1038/s42003-021-02410-9 (PMC8282876; doi:10.1038/s42003-021-02410-9)
Supplement: Supplementary file 3 — Description of Additional Supplementary Files [file 42003_2021_2410_MOESM3_ESM.pdf]

Description of Additional Supplementary Files

File Name: Supplementary Data 1
Description: All source data underlying the graphs presented in the main and supplementary Figures
