# Supplementary material for: Age-dependent appearance of SARS-CoV-2 entry sites in mouse chemosensory systems reflects COVID-19 anosmia-ageusia symptoms
Source: Commun Biol. 2021 Jul 15;4:880. doi: 10.1038/s42003-021-02410-9 (PMC8282876; doi:10.1038/s42003-021-02410-9)
Supplement: Supplementary file 5 — Reporting Summary [file 42003_2021_2410_MOESM5_ESM.pdf]

nature research

Corresponding author(s): Marie-Christine Broillet

Last updated by author(s): Jun 23, 2021

Reporting Summary

Nature Research wishes to improve the reproducibility of the work that we publish. This form provides structure for consistency and transparency in reporting. For further information on Nature Research policies, see our Editorial Policies and the Editorial Policy Checklist.

Statistics

For all statistical analyses, confirm that the following items are present in the figure legend, table legend, main text, or Methods section.

n/a	Confirmed	
☐	☒	The exact sample size (*n*) for each experimental group/condition, given as a discrete number and unit of measurement
☐	☒	A statement on whether measurements were taken from distinct samples or whether the same sample was measured repeatedly
☐	☒	The statistical test(s) used AND whether they are one- or two-sided<br>*Only common tests should be described solely by name; describe more complex techniques in the Methods section.*
☒	☐	A description of all covariates tested
☐	☒	A description of any assumptions or corrections, such as tests of normality and adjustment for multiple comparisons
☐	☒	A full description of the statistical parameters including central tendency (e.g. means) or other basic estimates (e.g. regression coefficient) AND variation (e.g. standard deviation) or associated estimates of uncertainty (e.g. confidence intervals)
☐	☒	For null hypothesis testing, the test statistic (e.g. *F*, *t*, *r*) with confidence intervals, effect sizes, degrees of freedom and *P* value noted<br>*Give P values as exact values whenever suitable.*
☒	☐	For Bayesian analysis, information on the choice of priors and Markov chain Monte Carlo settings
☒	☐	For hierarchical and complex designs, identification of the appropriate level for tests and full reporting of outcomes
☒	☐	Estimates of effect sizes (e.g. Cohen's *d*, Pearson's *r*), indicating how they were calculated

Our web collection on statistics for biologists contains articles on many of the points above.

Software and code

Policy information about availability of computer code

Data collection	RT-PCR and Western-blots were respectively aquired with transilluminator-UV chamber (biolabo) and FusionSolo (Viberand) internal softwares. For qRT-PCR, data acquisitions were done with the internal 7500 Fast Real-Time PCR software (Applied Biosystems). Values for RT-PCR, qRT-PCR and Western-blots were collected with Excel v14.7.7. Acquisitions of confocal SP5 were done using the Leica LAS v.AF software.
Data analysis	Semi-quantifications of gels were performed with NIH Fiji/ImageJ v1.53a. Confocal maximum projections were treated with Bitplane Imaris v7.2.1. Statistical analysis were performed with GraphPad Prism 8.4.3.

For manuscripts utilizing custom algorithms or software that are central to the research but not yet described in published literature, software must be made available to editors and reviewers. We strongly encourage code deposition in a community repository (e.g. GitHub). See the Nature Research guidelines for submitting code & software for further information.

Data

Policy information about availability of data

All manuscripts must include a data availability statement. This statement should provide the following information, where applicable:

- Accession codes, unique identifiers, or web links for publicly available datasets
- A list of figures that have associated raw data
- A description of any restrictions on data availability

All data generated or analysed during this study are included in this published article (and its supplementary information files).

Field-specific reporting

Please select the one below that is the best fit for your research. If you are not sure, read the appropriate sections before making your selection.

- [x] Life sciences
- [ ] Behavioural & social sciences
- [ ] Ecological, evolutionary & environmental sciences

For a reference copy of the document with all sections, see nature.com/documents/nr-reporting-summary-flat.pdf

Life sciences study design

All studies must disclose on these points even when the disclosure is negative.

Sample size	For RT-PCR and qRT-PCR, samples were obtained by pooling organs from 5-10 different mice at the indicated ages. For Western-blots, samples were obtained by pooling organs from 2-6 different mice at the indicated ages. Sample size was chosen on the basis of pilot experiments and according to previously reported publications done in the field. For histological experiments, the observed variability between individual acquisitions, at a given age, was modest and a minimum standard of triplicates was thus necessary.
Data exclusions	No data exclusion.
Replication	All findings were obtained from multiple and independent experiments. They were reliably reproduced.
Randomization	Equivalent sex-ratio were used for all experiments. For a given age, female and male mice were selected randomly.
Blinding	For all assays, the affiliated experimenter was aware of the conditions being tested (antibodies, organ of interest, ...). Nevertheless, they were conducted and analysed blinded to animal ages.

Reporting for specific materials, systems and methods

We require information from authors about some types of materials, experimental systems and methods used in many studies. Here, indicate whether each material, system or method listed is relevant to your study. If you are not sure if a list item applies to your research, read the appropriate section before selecting a response.

Materials & experimental systems

n/a	Involved in the study	
[ ]	[x]	Antibodies
[x]	[ ]	Eukaryotic cell lines
[x]	[ ]	Palaeontology and archaeology
[ ]	[x]	Animals and other organisms
[x]	[ ]	Human research participants
[x]	[ ]	Clinical data
[x]	[ ]	Dual use research of concern

Methods

n/a	Involved in the study	
[x]	[ ]	ChIP-seq
[x]	[ ]	Flow cytometry
[x]	[ ]	MRI-based neuroimaging

Antibodies

Antibodies used

1st Antibodies:
*Goat anti-ACE2; Invitrogen; 1:40; #PA5-47488, lot VJ3091799 / VJ3107190, Polyclonal. This antibody is widely used and has been validated by multiple previous publications that are listed on the manufacturer's website.

*Rabbit anti-TMPRSS2; abcam; 1:200; #ab109131, lot GR3343890-4, Monoclonal. This antibody is commonly used and has been validated by multiple previous publications that are listed on the manufacturer's website.

*Rabbit anti-CK18; Invitrogen; 1:50; #PA5-14263, lot VK3116392, Polyclonal. This antibody is commonly used and has been validated by multiple previous publications that are listed on the manufacturer's website.

*Rabbit anti-CK5; abcam; 1:160; #ab52635, lot GR3292032-3, Monoclonal. This antibody is commonly used and has been validated by multiple previous publications that are listed on the manufacturer's website.

*Rabbit anti-SOX2; Invitrogen; 1:200; #PA1-094, lot VI309496, Polyclonal. This antibody is commonly used and has been validated by multiple previous publications that are listed on the manufacturer's website.

*Rabbit anti-Galpha gust; Santa Cruz Biotechnology; 1:250; sc-395, lot F0711, Polyclonal. This antibody is widely used and has been validated by multiple previous publications that are listed on the manufacturer's website.

*Rabbit anti-CNGA2; Alomone Laboratories; 1:200; APC-045, lot APC045AN0302, Polyclonal. This antibody is widely used and has

been validated by multiple previous publications that are listed on the manufacturer's website.

*Rabbit anti-S100 beta; abcam; 1:500; #ab41548, lot GR22493-1, Polyclonal. This antibody is commonly used and has been validated by multiple previous publications that are listed on the manufacturer's website.

*Rabbit anti-PGCG; FabGennix; 1:250; #PGCG-701 AP, lot A255.PB2-4.AP, Polyclonal. This antibody is commonly used and has been validated by multiple previous publications that are listed on the manufacturer's website.

*Rabbit anti-gamma Tubulin; abcam; 1:250; #ab179503, lot GR208789-13, Monoclonal. This antibody is commonly used and has been validated by multiple previous publications that are listed on the manufacturer's website.

*Rabbit anti-Actin; Merck; 1:2500; #A2066, lot 069M4762V, Polyclonal. This antibody is commonly used and has been validated by multiple previous publications that are listed on the manufacturer's website.

2nd Antibodies:
*Alexa Fluor Plus 647-conjugated, Donkey anti-Goat; Invitrogen; 1:200; #A32849, lot VH306860, Polyclonal. This antibody has been validated in several previous publications that are listed on the manufacturer's website.

*Cy3-conjugated, Donkey anti-Rabbit; Jackson ImmunoResearch; 1:200; #711-165-152, lot 151285, Polyclonal. This antibody has been validated in several previous publications that are listed on the manufacturer's website.

*Horseradish Peroxidase-conjugated, Donkey anti-Goat; Jackson ImmunoResearch; 1:4000; #705-035-003, lot 143452, Polyclonal. This antibody has been validated in several previous publications that are listed on the manufacturer's website.

*Horseradish Peroxidase-conjugated, Goat anti-Rabbit; Jackson ImmunoResearch; 1:10000; #111-035-003, lot 146500, Polyclonal. This antibody has been validated in several previous publications that are listed on the manufacturer's website.

Validation

All antibodies are commercial and have been validated. Informations are available on the manufacturers' publicly accessible datasheets.

Animals and other organisms

Policy information about studies involving animals; ARRIVE guidelines recommended for reporting animal research

Laboratory animals

Adult (4-20 M) and pups (0.5 M) male and female C57BL/6 (Mus musculus) and OMP-GFP mice were used. In the gene-targeted mouse strain OMP-GFP , the GFP is used as a histological reporter of mature olfactory sensory neurons expressed under the control of the olfactory marker protein (OMP) promoter.

Wild animals

The study did not involve wild animals.

Field-collected samples

The study did not involve samples collected from the field.

Ethics oversight

The animal experimental procedures were in accordance with the Swiss legislation and approved by the EXPANIM committee of the Lemanique Animal Facility Network and the veterinary authority of the Canton de Vaud (SCAV).

Note that full information on the approval of the study protocol must also be provided in the manuscript.
